# Drivers and hazards of consumption of unpasteurised bovine milk and milk products in high-income countries

**DOI:** 10.7717/peerj.13426

**Published:** 2022-05-16

**Authors:** Joanna N. de Klerk, Philip A. Robinson

**Affiliations:** 1 School of Life Sciences, University of Warwick, Coventry, United Kingdom; 2 Department of Animal Health, Behaviour and Welfare, Harper Adams University, Newport, Shropshire, United Kingdom

**Keywords:** Milk, Unpasteurised, Zoonoses, Hazards, Infectious diseases, Dairy, Bovine, Global, Drivers, Raw milk

## Abstract

**Introduction:**

The consumption of dairy products contributes to health, nutrition, and livelihoods globally. However, dairy products do not come without microbiological food safety risks for consumers. Despite this risk, common hygiene measures in high-income countries, particularly pasteurisation, ensures that milk is safe, and is indeed frequently mandated by law. Nevertheless, over the past two decades, there has been a global increase in the number of consumers in high-income developed countries actively seeking out unpasteurised milk in liquid and product forms for perceived nutritional and health benefits, and improved taste. The often-anecdotal claims upon which consumers make such choices are not all supported by scientific evidence; however, some recent research studies have investigated (and in some cases demonstrated) the positive impact of unpasteurised milk consumption on the prevalence of asthma, atopy, rectal cancer and respiratory illness.

**Methods:**

To investigate the significance of unpasteurised milk and milk product consumption for human health in high-income countries, outbreak data between the years 2000 and 2018 were obtained for the United States of America, Canada, the European Union, the United Kingdom, Japan, New Zealand and Australia, which were then categorized into three World Health Organisation subregions: AMR A, EUR A and WPR A. Outbreak dynamic variables such as pathogens, the place of consumption, numbers of outbreaks and deaths per million capita, the average number of cases per outbreak and regulations were described and analysed using R Studio. To provide an overview of unpasteurised milk-related disease outbreaks, a rapid evidence review was also undertaken to establish an overview of what is known in the current literature about hazards and drivers of consumption.

**Results:**

Foodborne outbreaks associated with unpasteurised dairy consumption have risen in high-income countries over the period 2000 to 2018, with *Campylobacter* spp. being the most common aetiological agent responsible, followed by *Escherichia coli* and *Salmonella* spp. The most common places of consumption are on farms or in households, indicating individuals choose to drink unpasteurised milk, rather than a widespread distribution of the product, for example, at social events and in schools. Further study is needed to better understand contributing factors, such as cultural differences in the consumption of dairy products.

**Conclusion:**

There are several observable health benefits linked to consuming raw milk, but outbreaks associated with unpasteurised milk and milk products are on the rise. It cannot be definitively concluded whether the benefits outweigh the risks, and ultimately the decision lies with the individual consumer. Nevertheless, many countries have regulations in place to protect consumer health, acknowledging the definite risks to human health that unpasteurised dairy foods may pose, particularly from microbial hazards.

## Introduction

Approximately 4% of the global foodborne disease burden, and 12% of the animal source disease burden is due to dairy products (Grace, Wu & Havelaar, 2020). While this encompasses both unpasteurised and pasteurised dairy products, unpasteurised milk presents a number of additional hazards, and the size and frequency of disease outbreaks in people may be influenced by these hazards. Unpasteurised milk is defined as “milk produced by the secretion of the mammary gland of farmed animals that has not been heated to more than 40 °C or undergone any treatment that has an equivalent effect” (European Food Safety Authority (EFSA), 2015). It is also known as raw milk or raw drinking milk (RDM) when liquid, and can be collected from any mammal. Most commonly, milk from cows, goats, sheep, buffaloes, or camels is produced for consumption. The research presented in this paper solely focuses on cow milk (both in liquid and product forms), as bovines produce 96% of the world’s milk (Grace, Wu & Havelaar, 2020).

Raw milk consumption is often an emotive issue which splits opinion between state regulatory authorities, veterinarians, farmers and consumers, and also between research scientists. The consumption of dairy products plays an important and positive role in global health and nutrition, as well as supporting livelihoods, but there are also associated hazards with dairy consumption in the form of foodborne diseases when unpasteurised dairy products are consumed. The clinical symptoms associated with the most common unpasteurized milk pathogens are shown in Table 1 (Oliver et al., 2011). However, hygiene control measures, such as product pasteurisation, a herd health focus, and meticulous attention to milking practices aimed at reducing milk contamination reduce the risk of dairy-related foodborne disease. In many high-income countries, these practices have become legal requirements to ensure the provision of safe milk for the end consumer, resulting in dairy foodborne outbreaks contributing to only 2–6% of all foodborne diseases in high-income countries (Claeys et al., 2013). Conversely, many low- and middle-income countries do not have the same hygienic closed milking systems that contribute to food safety, and lack the financial capacity for all the milk produced to be pasteurised. Therefore, dairy-related foodborne outbreaks are more frequent than in high-income countries (Grace, Wu & Havelaar, 2020).

**Table 1:** An overview of the clinical symptoms associated with the most common pathogens associated with dairy foodborne diseases (Oliver et al., 2011).

Pathogen	Clinical symptoms
*Campylobacter jejuni*	Diarrhoea (occasionally haemorrhagic), fever, dizziness, vomiting, gastrointestinal pain
*Listeria monocytogenes*	Diarrhoea, flu-like symptoms, miscarriages, meningitis
*Salmonella* spp*.*	Vomiting, headache, diarrhoea, flu-like symptoms, gastrointestinal pain
*Staphylococcus aureus*	Vomiting, diarrhoea
*Shiga toxin-producing Escherichia coli*	Haemorrhagic diarrhoea, gastrointestinal pain, vomiting, haemolytic uraemic syndrome (HUS), kidney failure, fever, death
*Vibrio parahaemolyticus*	Vomiting, diarrhoea, gastrointestinal pain, fever, chills
*Yersina enterocolitica*	Fever, diarrhoea, vomiting, gastrointestinal pain

**Figure 1:**
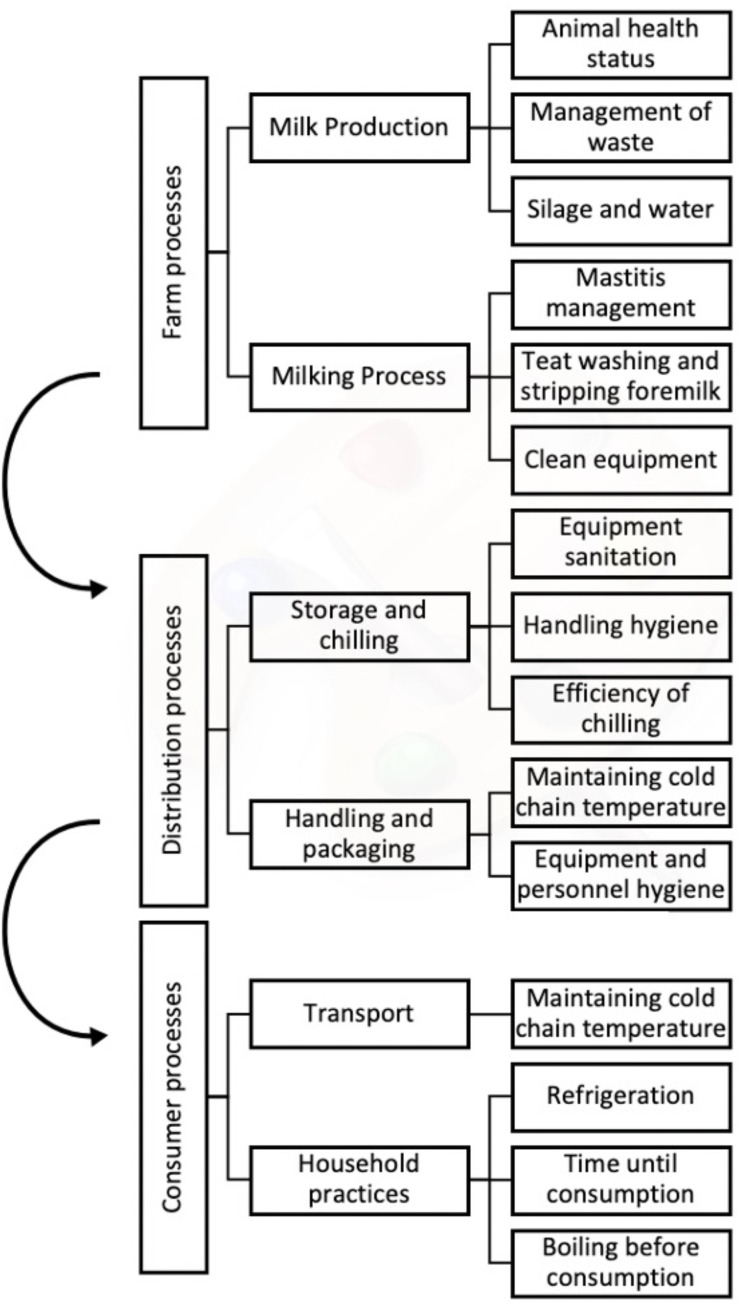
Flow diagram to highlight the processes which enable hazards, such as faecal contamination, environmental contamination, and microbial growth, to affect the milk.

Pathogenic microbes can enter the milk from faecal contamination, environmental contamination or disease of the cow. Multiple factors impact the risk associated with the journey of the milk from the animal to the end consumer. This risk pathway is demonstrated in Fig. 1. Since the pathway has many potential points of entry for hazards, even with the most diligent of producers, distributors, and consumers, the final product could still be hazardous without further controls, and pasteurisation helps to protect consumer safety. Pasteurisation has also further facilitated the ease of distribution, handling and shelf-life of milk for human consumption. Despite the benefits, pasteurisation is not perfect. Experimentally, *Clostridium botulinum* spores, *Bacillus cereus* spores, *E. coli* and *Staphylococcus aureus* have been shown to survive the pasteurisation process, which is why refrigeration below 7 °C is important (Office for Risk Assessment & Research (BuRO), 2017; European Food Safety Authority (EFSA), 2011). Temperature control contributes significantly to whether the microbes can lead to disease in the consumer. If the milk is not kept cold at refrigeration temperatures (2–8 °C), this is the point in the risk pathway at which the most rapid growth of pathogens occurs (Food Standards Australia New Zealand (FSANZ), 2009).

Unpasteurised milk contains lactic acid bacteria, which naturally inhibit some pathogenic bacteria, but pasteurisation destroys these, resulting in a lack of natural inhibition within the milk (European Food Safety Authority (EFSA), 2013). However, the effects of lactic acid bacteria are insufficient to make unpasteurised milk safe, as some bacteria have a very low infective dose, and small numbers can still make the milk hazardous for consumption. In addition, enzymes associated with the lactic acid bacteria, which have acidifying and proteolytic abilities, are limited in their effects with the refrigerated temperatures at which milk is stored (European Food Safety Authority (EFSA), 2011).

Despite unpasteurised milk being accountable for a higher percentage of reported dairy-borne food outbreaks than pasteurised milk (*e.g.*, in the USA—Whitehead & Lake, 2018), there has been an increased production and consumption trend globally. In the United Kingdom (UK), the volume of production of unpasteurised milk destined for drinking increased from 610,000L in 2012 to 3.2 million litres in 2017 (Food Standards Agency (FSA), 2018a). In addition, in 2012 only 3% of the UK population consumed unpasteurised milk regularly, whereas that figure had increased to 10% by 2018 (Food Standards Agency (FSA), 2018a), demonstrating the increasing popularity of the practice. This is likely to be due to many observed or perceived health benefits, which will be further explored in this article.

Regulation and consumption of unpasteurised milk have been controversial since the early 20th century (Linn, 2019), and there have been extensive peer-reviewed analyses on the benefits, risks and regulations (*e.g.*, Draper & Green, 2002; Enticott, 2003; West, 2008; Knutson et al., 2010; Dunn, 2011; MacDonald et al., 2011; Buzby et al., 2013; Paxson & Helmreich, 2014; Knezevic, 2016; Rahn, Gollust & Tang, 2017; Willis et al., 2018; Berge & Baars, 2020; Baars et al., 2021). In this way, when the public is confronted with a large body of conflicting information (such as whether unpasteurised milk is safe, beneficial, or hazardous), and if selectively presented, there is the potential to yield significant differences in opinion to that of scientific experts (Angulo, Lejeune & Rajala-Schultz, 2009). Therefore, many countries have adopted a restrictive position on allowing the sale of unpasteurised milk for consumption, and have protected the public from the associated hazards by controlling or banning the sale of unpasteurised milk through legislation.

The aim of the research presented in this paper was to investigate whether consumption of unpasteurised milk and milk products is a problem within high-income countries, and to discuss whether further action needs to be in place to prevent or reduce dairy-related foodborne outbreaks. Therefore, based on a rapid evidence review and the sourcing of primary outbreak data from indicative high-income countries around the world, the research explored the following research questions: Why do people consume unpasteurised liquid milk and milk products in high-income countries? What are the biological hazards associated with unpasteurised milk consumption? What can be done to decrease the risk of foodborne outbreaks associated with unpasteurised milk consumption?

## Materials & Methods

### Rapid evidence review

A rapid evidence review (also known as an RER, rapid review, or rapid evidence assessment) was undertaken to establish the drivers, hazards and regulations currently highlighted in publications, and to provide a relevant and broad summary of the recent literature on unpasteurised milk consumption. While there is no agreed definition or methodology for RERs (Haby et al., 2016), the Cochrane Rapid Reviews Methods Group methodology was used as a guideline (Cochrane, 2020). It must be noted that it was not an exhaustive literature review, as a RER aims to provide an overview assessment about what is already known about a social, practice or policy issue, however conducted over a shorter timeframe than a systematic review (Grant & Booth, 2009).

The procedure for the review began by identifying search terms related to unpasteurised milk consumption and biological hazard control. Then a primary database of evidence from the published literature was developed based on four categories: “drivers”, “hazards”, “policy” and “control”. The search function “AND” with the search term “unpasteurised milk” was used to identify articles which were in each of the categories, and variations of the keywords were also combined by using the “OR” search function, such as “benefits”, “pathogens”, “regulations”, “safety”, “alternatives” and “raw milk”. ‘Grey’ literature (research reports, working papers, policy documents etc.) was identified using the same search terms on Google and websites belonging to government departments responsible for foodborne outbreaks for the countries selected and included in this review. The first 20 pages of search results from all search engines and websites used, where available, were then scanned.

This review comprised several inclusion criteria. Literature published before the year 2000 was excluded, except for publications relating to policy and regulation, which were required to be more recently published than 2015. Articles that were not focussed on bovine milk or milk consumption in low- or middle-income countries were also excluded. Titles were first examined to exclude literature obviously irrelevant to the research. Of the remaining articles, abstracts were then screened, followed by full papers or websites being read. Articles in alternative languages to English were included if the abstract was available in English. Citations of included articles were then scanned for relevance. Articles were then categorised into the four previously mentioned categories, and cross-referenced by country of focus of the article.

### Primary outbreak data

Epidemiological data focussing on dairy-related foodborne outbreaks between the years 2000 and 2018 were obtained through direct communication with public health and food safety authorities in indicative high-income countries around the world, freely accessible open access data on the internet, and report publications produced by the USA’s CDC National Outbreak Reporting System (NORS), Public Health Agency of Canada, Public Health England (PHE), the European Food Safety Authority (EFSA), New Zealand Food Safety, Food Safety Australia New Zealand, New Zealand’s ESR Public Health Surveillance and Japan’s Ministry of Health. For publicly available data where a search could be performed from a drop-down menu or filter of food vehicles, search terms included “raw” or “unpasteurised” as well as all drinking milk and milk products, such as, cheese, ice cream or cream. For data not openly available to the public, all unpasteurised dairy products were requested. Products that were made with unpasteurised milk but then cooked, were removed from the dataset. Only bovine milk was analysed, so unpasteurised products from other food-producing animals were also excluded. Some food products had no outbreaks recorded, such as unpasteurised butter, yoghurt or fermented raw milk products.

Countries for analysis were chosen from three of the fourteen World Health Organisation’s subregion groupings (World Health Organization (WHO), 2015). The three subregions were defined as having “very low child and adult mortality” and contain most of the countries classified by the World Bank as high-income, where the gross national income per capita exceeds $12,696 (World Bank, 2021). In addition to this, they are the opposite of those studied in Grace et al.’s research on foodborne diseases from milk in developing countries (Grace, Wu & Havelaar, 2020). Region “AMR A” contained the high-income countries USA and Canada. Cuba was removed from this group due to being classified as upper middle income, not high income, by the World Bank. “EUR A” contained Austria, Belgium, Croatia, Cyprus, Czech Republic, Denmark, Finland, France, Germany, Greece, Ireland, Italy, Luxembourg, Malta, Netherlands, Portugal, Slovenia, Spain, Sweden, and the UK. However, Andorra, Israel, Iceland, Monaco, Norway, San Marino, and Switzerland are not members of the European Union, resulting in them having their own disease reporting agencies, from which data was requested but could not be obtained. Therefore, they were excluded from the analysis. In addition to this, some countries included in the “EUR A” group did not report any foodborne outbreaks to the EFSA, and therefore did not contribute to the analysis. These included Belgium, Cyprus, Czech Republic, Greece, Luxemburg, Malta, Portugal, Slovenia, and Spain. Finally, “WPR A” contained Australia, Japan, and New Zealand. However, Brunei Darussalam and Singapore were excluded from the analysis as no data could be found for them. The outcome variable used was the number of outbreaks, not the number of cases. An outbreak was defined as “two or more cases of similar illness associated with a common exposure” (Centers for Disease Control and Prevention (CDC), 2017). Variables were described and graphically analysed using Microsoft Excel (version 16.52, Microsoft Corporation, Redmond, USA) and R Studio (version 1.3.1093, https://www.rstudio.com/).

## Results

### Rapid evidence review

Of the published literature reviewed in this study, there were found to be significantly more articles on the biological hazards of unpasteurised milk consumption, rather than the drivers or benefits of consumption. Limited literature was available exploring alternatives to pasteurisation (two peer-reviewed articles), and the remainder of the literature being ‘grey’ literature. Information on regulations related to unpasteurised milk was obtained from government publications, as no peer-reviewed publications could be found which reviewed the specific regulations of the countries, nor exploring how the differences in regulations might impact the dairy foodborne outbreaks.

### Perceived and demonstrable benefits from unpasteurised milk consumption

A 2018 survey for the UK’s Food Standards Agency (FSA) determined that the two most common reasons for drinking unpasteurised milk were the belief that it had a higher nutritional content (59% of consumers identified this as a reason), and that it was easier to digest (40%). However, there were additional reasons or beliefs, including: an improved taste (29%), prevention of asthma and allergies (28%), less environmental impact (25%) and supporting local farmers (16%) (Food Standards Agency (FSA), 2018b). In addition to the reasons provided in this survey, culture has also been suggested to play an important role in the consumption of unpasteurised milk. Many older consumers have been reared on unpasteurised milk and have the mindset that it has not harmed them in the past, so there is unlikely to be future harm; consumption is part of their lifetime tradition (Enticott, 2003). As a result of this combination of factors, unpasteurised milk is often therefore perceived in a positive light by consumers. In the 2018 FSA survey, for example, it was recorded that 67% of conversations on the topic of unpasteurised milk were positive, 23% were neutral, and only 10% were negative (Food Standards Agency (FSA), 2018b). This was the only study available from the rapid evidence review which quantified consumer opinions, and therefore reasoning for consumption in other regions of the world may differ.

In terms of benefits investigated through scientific analysis, MacDonald et al. (2011) reviewed nutritional changes during pasteurisation, and concluded that vitamins B1, B2, B12, C, E and folate decreased, whereas vitamin A increased. However, except for vitamin B2, the levels are so low that these changes make an insignificant contribution to dietary composition (MacDonald et al., 2011). The effects of pasteurisation on vitamin D were not explored in this review, as bovine milk is deficient in vitamin D, resulting in pasteurised milk often being fortified with it (MacDonald et al., 2011). Nonetheless, milk can differ significantly from one batch to the next due to factors such as cow breed, season, vitamin concentrations in the feed, feed composition, country of origin and milking frequency. In addition, methods to measure water-soluble vitamins, such as vitamin C and vitamin B2, have been shown to be unreliable, and therefore it is difficult to conclude that these recorded nutritional differences are accurate (MacDonald et al., 2011).

Although there may be negligible nutritional differences between pasteurised and unpasteurised milk, there has now been reproducible evidence that unpasteurised milk consumption is significantly associated with immunological benefits such as protection against atopy, asthma, and respiratory illnesses (Brick et al., 2020). For example, the GABRIEL study included 10,000 children across three countries (Ege et al., 2011; Loss et al., 2011; Illi et al., 2012; Horak et al., 2014); the PARSIFAL study included 15,000 children across five countries (Bieli et al., 2007; Waser et al., 2007); and the PASTURE prospective birth cohort study monitored 900 children across six countries until age six (Schaub et al., 2009; Loss et al., 2012; Depner et al., 2013; Lluis et al., 2014; Loss et al., 2015; Brick et al., 2016). These studies investigated the “farm effect”, where children exposed to a farming environment had a lower prevalence of allergic conditions. More recently, the Agricultural Lung Health study included a large cohort of 3,000 farmers and spouses in the United States, and concluded that childhood consumption of unpasteurised milk improved lung function in adult life, and protected against later development of atopy and asthma (House et al., 2017; Wyss et al., 2018).

Several different milk constituents have been suggested in the literature as contributors to these benefits: whey proteins, polyunsaturated fatty acids, microRNA, and oligosaccharides (Abbring et al., 2019a; Brick et al., 2020); however, their effects on the immune system are not yet fully understood in humans. Nevertheless, whey proteins appear to be involved in the allergenic processes of mice, resulting in a less allergenic reaction when sensitised with raw milk than pasteurised milk, hypothesised by a change in allergen uptake or an alteration in environment which favours a reduced response to allergens (Abbring et al., 2019b). There has also been a murine study which has demonstrated activation of T-cell related genes which could be responsible for increased allergen tolerance (Abbring et al., 2019c). Finally, it has also been suggested that exposure to the more abundant microbes in unpasteurised milk consumed during the development of the immune system may prevent hypersensitivity to allergens (MacDonald et al., 2011).

These studies have provided some evidence of the protective benefits of unpasteurised milk consumption on allergies, however there may have been farm-related confounders at play, since exposure to microbes from many sources is common in a farm-living lifestyle (MacDonald et al., 2011). Nonetheless, one study of children who consumed unpasteurised milk, and lived in rural areas but *not* on a farm, demonstrated a decreased odds ratio of developing asthma (Brick et al., 2020).

Concerns about the consumption of unpasteurised milk being linked to the subsequent risk of cancer have been raised (Sellers et al., 2008). However, one comprehensive study including 41,836 women, explored 2,379 types of cancer, and concluded that consumption of unpasteurised milk was not significantly associated with increased or decreased risk of cancer, except for rectal cancer, where there was a decreased relative risk for developing rectal cancer when unpasteurised milk was consumed as an adult (RR = 0.2, 95% CI [0.1–0.69]) (Sellers et al., 2008). However, the sample size for this type of cancer was small.

Another significant perceived health benefit which motivates the consumption of unpasteurised milk is the claim that it is easier to digest. Around 75% of people worldwide have some degree of lactose intolerance, resulting in gastrointestinal symptoms after consuming dairy products. Unpasteurised milk contains beneficial bacteria, such as *Lactobacillus acidophilus*, that produce lactase enzymes to aid in the digestion of lactose (MacDonald et al., 2011), but these enzymes are destroyed during the pasteurisation process. Unpasteurised milk and dairy products have been associated with the growth and increased abundance of *Lactobacillus* in the gut microbiome compared to controls in an observational study (Butler et al., 2020). Despite these potential benefits for milk digestion, Mummah et al. (2014) failed to demonstrate a link between unpasteurised milk consumption and an improvement of symptoms related to lactose intolerance.

### Biological hazards found in unpasteurised milk

Even though there may be benefits from drinking unpasteurised milk, there are also many hazards, and the negatives may outweigh the positives from a public health perspective (Angulo, Lejeune & Rajala-Schultz, 2009). In high-income countries, there are usually efficient communication systems to educate consumers on the basics of food safety, and while consumers may still remain unaware of specific hazards, others may choose to continue to drink unpasteurised milk and eat unpasteurised dairy products because their own risk assessment and priorities mean that the benefits outweigh the negatives.

The scientific literature describes multiple hazards. Dairy hazards can be biological (bacteria, viruses and parasites), chemical (heavy metals, mycotoxins and industrial chemicals), physical (stones, fragments of glass or metal) or allergenic (Grace, Wu & Havelaar, 2020). However, the RER only focussed on the biological hazards of unpasteurised bovine milk and milk products. Milk from other species was not included, as different pathogens could be involved compared to bovine. For example, goats’ milk carries the risk of tick-borne encephalitis virus, which does not appear to be a problem in cows’ milk (European Food Safety Authority (EFSA), 2013).

*Campylobacter* spp., Shiga-toxin producing *Escherichia coli*, *Salmonella* spp., *Cryptosporidium parvum, Bacillus cereus, Staphylococcus aureus, Yersina pseudotuberculosis*, and *Toxoplasma gondii* are all recognised pathogens for dairy (European Food Safety Authority (EFSA), 2015; Li et al., 2019). However, others have been occasionally cited. *Listeria monocytogenes* appears to be more commonly associated with cheeses, rather than liquid milk (De Buyser et al., 2001). *Coxiella burnetii* has also been proven to have a hypothetical risk of transmission in unpasteurised milk, although few outbreaks have been recorded (Gale et al., 2015). Methicillin-resistant *Staphylococcus aureus* (MRSA) also provides a possible threat. Even though it has not been associated with foodborne outbreaks in dairy, there have been increasing numbers of reports of isolation from bulk-tank milk and dairy farms in the EU (European Food Safety Authority (EFSA), 2015).

*Mycobacterium bovis* and *Brucella abortus* in raw milk do not appear to be major concerns in modern times in developed countries, due to disease eradication programmes and pasteurisation, they are considered low threats (Food Standards Australia New Zealand (FSANZ), 2009). However, Robinson (2019) has argued that the risk to humans may be under-appreciated in developed countries with endemic bovine tuberculosis, and outbreaks linked to unpasteurised milk consumption have occurred associated with both diseases (*e.g.*, *M. bovis*—Doran et al., 2009; *B. abortus* RB51 vaccine-associated—Sfeir, 2018).

Dairy foodborne outbreaks receive frequent attention from the media, and many media articles were retrieved in the RER. However, the number of cases reported are likely to only be only a small proportion of the number of cases associated with milk. It has been estimated that for every illness associated with an outbreak, an additional 26–100 illnesses are likely to occur, depending on the pathogen (Robinson, Scheftel & Smith, 2014). Conversely, it is important to consider that outbreaks associated with raw milk may be subject to a detection bias compared to store-bought products, as the consumer is more easily able to recall actively sought-after niche commodities (Berge & Baars, 2020).

With heat treatment, such as ultra-high temperature (UHT) treated milk, pasteurisation, or boiling, biological hazards can be reduced. A study by Loss et al. (2011) explored the percentage of milk samples with detectable microbes, categorised by the heat treatment they had received. Considerably more samples of raw milk contained microbes, such as micrococci, *Lactobacilli*, yeasts, and psychotropic bacteria (which most bacterial milk hazards discussed in this paper can be categorised into), compared to milk which had received some form of heat treatment. A study by Willis et al. (2018) looked at 770 samples of raw milk on retail shelves and found that 41% were unsatisfactory due to elevated aerobic colony counts or detectable levels of injurious bacteria. However, it has been demonstrated in Germany that it is feasible to produce raw milk with the same hygiene level as pasteurised milk, resulting in the possible equalisation of the microbial risk (Berge & Baars, 2020). Nevertheless, the presence of microbes does not necessarily correlate with the causation of disease. It is often the case that certain conditions, such as a break in the cold chain, have provided opportunities for microbes to multiply and reach a level at which they are harmful. However, not all microbes fall into this category. *Campylobacter* spp., for example, enters the milk from faecal contamination during the milking process, and cannot multiply in raw milk regardless of the environment. Also, only a very low count of *Campylobacter* spp. is required to cause an illness, so the cold chain is irrelevant for control of this particular bacterium (BfR, 2016).

### Policy on acquisition of unpasteurised milk

Table 2 outlines the current regulations associated with unpasteurised milk availability in high-income countries available from government literature. For many countries, this includes meeting hygiene indicators as well as retail constrictions.

**Table 2:** An overview of the regulations associated with the production and sale of unpasteurised milk in a selection of the high-income countries associated with the WHO regions of interest in this article (updated August 2021).

Country	Regulations
United States of America	All member states have adopted the “Grade A Pasteurised Milk Ordinance” (PMO) which is a set of standards for the production, processing, and packaging of milk. Each member state determines if the sale of unpasteurised milk is illegal. As of 2021, 23 states do not allow the sale of unpasteurised cows’ milk in retail stores, at the farm, or through off-farm sales (Farm-to-Consumer Legal Defense Fund (FTCLDF), 2021), whereas the remaining states allow retail, on-farm and/or off-farm sales, subject to individual state laws and regulations on hygiene, testing, and licencing.
Canada	The sale of unpasteurised milk has been illegal since 1991, however, soft, and semi-soft cheeses made from unpasteurised milk are allowed, with the requirement of the label “made from raw or unpasteurised milk”.
United Kingdom	The sale of unpasteurised milk in Scotland is banned, however it is legal in England, Wales, and Northern Ireland. Unpasteurised milk can only be sold at the farm gate, at registered farmers’ markets, milk round or similar distributors, direct online sales, and at farm vending machines. Milk must be labelled with a health warning, be from a farm which is hygiene inspected twice a year and collected from tuberculosis and brucellosis-free animals. It must also meet the standards of a total bacteria count of under 20,000 cfu per ml and under 100 cfu per ml of coliforms.
Ireland	Farms which sell more than 30 litres of unpasteurised milk per week or sell further away than a 20 km radius of the farm must register with the DAFM. Animals must have a somatic cell count (SCC) less than 200,000 per ml and are inspected twice yearly.
European Union	Member states can determine their own laws regarding the legal sale of unpasteurised milk, as well as set their own requirements for hygiene and quality testing.
Switzerland	Unpasteurised milk can be sold for human consumption, but it is illegal to advertise. Milk must be sampled twice monthly and have a somatic cell count (SCC) of below 350,000 per ml.
Australia	The sale of unpasteurised milk and milk products for human consumption is illegal. In addition to this, in Victoria, it is also illegal to package or deliver unpasteurised milk.
New Zealand	Unpasteurised milk can only be sold directly to the consumer from the farm, or home delivery by the farmers. Unpasteurised milk is subject to requirements laid out in a regulated control scheme (RCS).
Japan	Unpasteurised milk is available in Japan at facilities which have received special permission. As of 2021, only one farm produces unpasteurised milk. Unpasteurised milk must be clearly labelled, have a total bacterial count (TBC) of under 30,000 cfu per ml, and be negative of coliforms.
Singapore	The sale of unpasteurised milk for human consumption is prohibited.

Despite the ban on sale of unpasteurized milk in some countries, raw milk advocates may find ways to circumvent the regulations (Buzby et al., 2013). For example, cow-share schemes have provided ways in which consumers can acquire milk without buying it, instead owning a share of the cows producing the milk. Through such schemes, consumers pay for the upkeep, milking, and general care of the cows, and, in return, receive unpasteurised milk from “their” animals. Another common method of acquisition is through the purchase of “pet milk”. In some countries, the sale of unpasteurised milk for pets is allowed, if it is clearly labelled “not for human consumption”. However, this may not end up being fed to pets, but may instead be consumed by people (Angulo, Lejeune & Rajala-Schultz, 2009).

### Primary outbreak data

Due to the geographical differences between countries in terms of legislation, management of milk, and level of surveillance, which consequently impacted the number of outbreaks and available data for those outbreaks, the results in this section are only indicative of trends.

**Figure 2:**
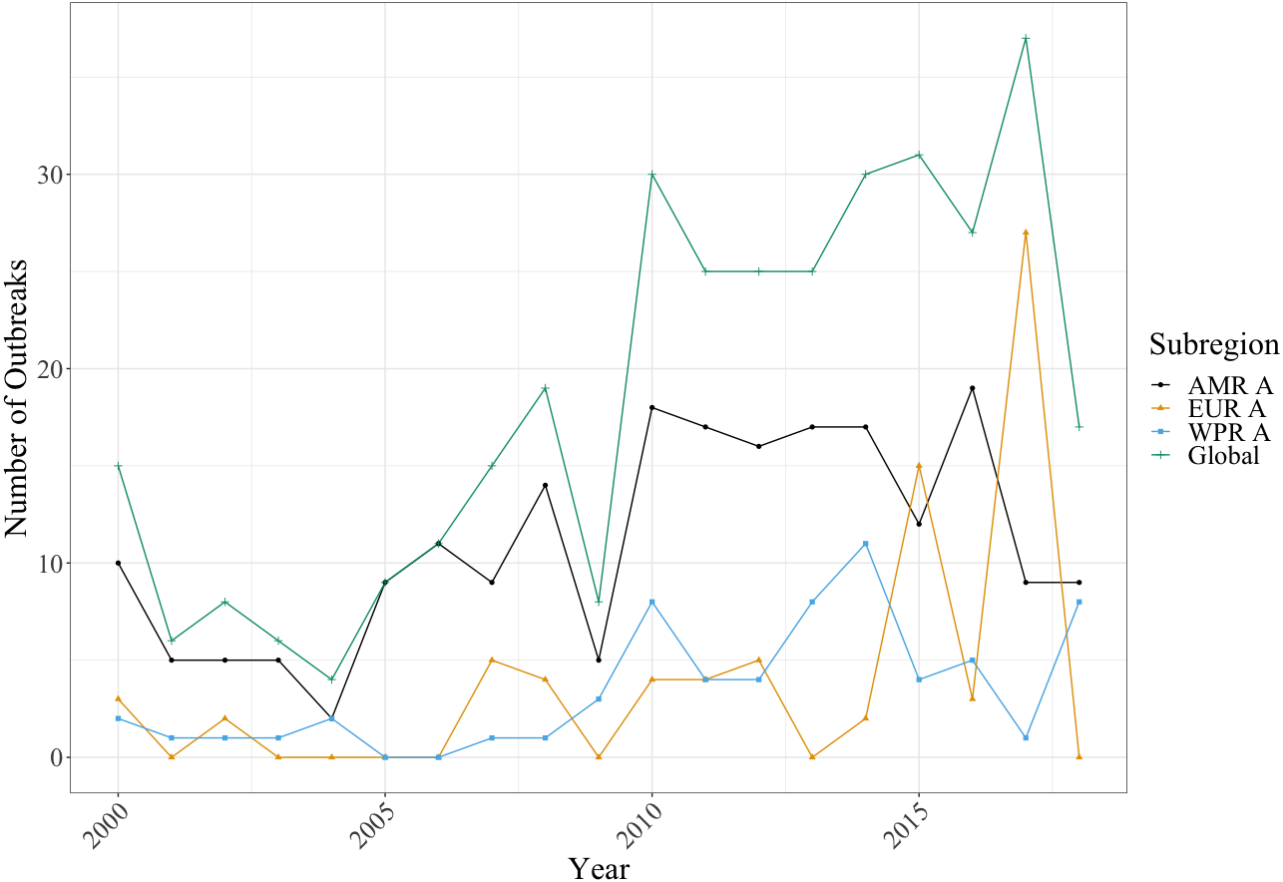
Line graph showing the number of outbreaks per WHO subregion between the year 2000 and 2018. CDC NORS data for AMR A in 2009 contained data quality issues so there was a decrease by almost 50% on the previous 5-year average contributing to the global dip in that year. Further data by country is available in Table S1.

As shown in Fig. 2, the number of outbreaks associated with unpasteurised milk consumption have been on the rise over the past two decades. Pearson’s correlation coefficient for the high-income countries’ number of outbreaks over time indicated a strong, positive correlation (*r* = 0.79). This trend was also reflected across all WHO subregions (AMR A, *r* = 0.57; EUR A, *r* = 0.46; WPR A, *r* = 0.62).

Whilst the USA was the country with the most outbreaks related to raw milk consumption between the years 2000 to 2018 (204 out of 343), the number of outbreaks per million capita was only 0.61. The country with the greatest number of outbreaks per million capita was New Zealand, at 12.32 outbreaks, followed by Finland, at 0.90 outbreaks. The countries with the least number of outbreaks per million capita were France and Japan, at 0.02, although it must be noted that Belgium, Cyprus, Czech Republic, Greece, Luxemburg, Malta, Portugal, Slovenia, and Spain recorded no outbreaks within the survey period, and it is unknown whether this was due to there being no outbreaks, or simply a failure to report.

The average number of cases per outbreak ranged from three, in Austria, Croatia and Ireland, to 30.33 in Japan. The only countries reported to experience deaths from outbreaks associated with unpasteurised milk consumption were the UK, the USA and Canada, at a rate of 0.015, 0.015 and 0.026 deaths per million capita respectively. Further data can be found in Table 3.

**Table 3:** A table displaying the outbreaks, cases per outbreak, and deaths per outbreak per capita in each country which contributed outbreak data to this research. Belgium, Cyprus, Czech Republic, Greece, Luxemburg, Malta, Portugal, Slovenia, and Spain are all required to report cases to the EFSA, however, did not report any dairy-related foodborne disease for the study period of 2000 to 2018.

Country	Population (million)	Outbreaks	Outbreaks per million	Cases total	Cases per outbreak	Deaths total	Deaths per million
Australia	25.85	6	0.23	80	13.33	0	0
Austria	9.067	2	0.22	6	3.00	0	0
Canada	37.74	5	0.13	59	11.80	1	0.027
Croatia	4.07	1	0.25	3	3.00	0	0
Denmark	5.82	4	0.69	95	23.75	0	0
Finland	5.55	5	0.90	104	20.80	0	0
France	65.45	1	0.02	29	29.00	0	0
Germany	83.12	34	0.41	522	15.35	0	0
Ireland	4.76	2	0.42	6	3.00	0	0
Japan	126.01	3	0.02	91	30.33	0	0
Netherlands	17.18	4	0.23	68	17.00	0	0
New Zealand	4.87	60	12.32	257	4.28	0	0
Sweden	10.42	1	0.10	13	13.00	0	0
UK	68.31	11	0.16	136	12.36	1	0.015
USA	332.66	204	0.61	2990	14.66	5	0.015

Unpasteurised milk was the food vehicle for 89.9% of the high-income country outbreaks between the years 2000 and 2018, whereas cheese accounted for 9.2% and ice-cream 0.9%.

**Figure 3:**
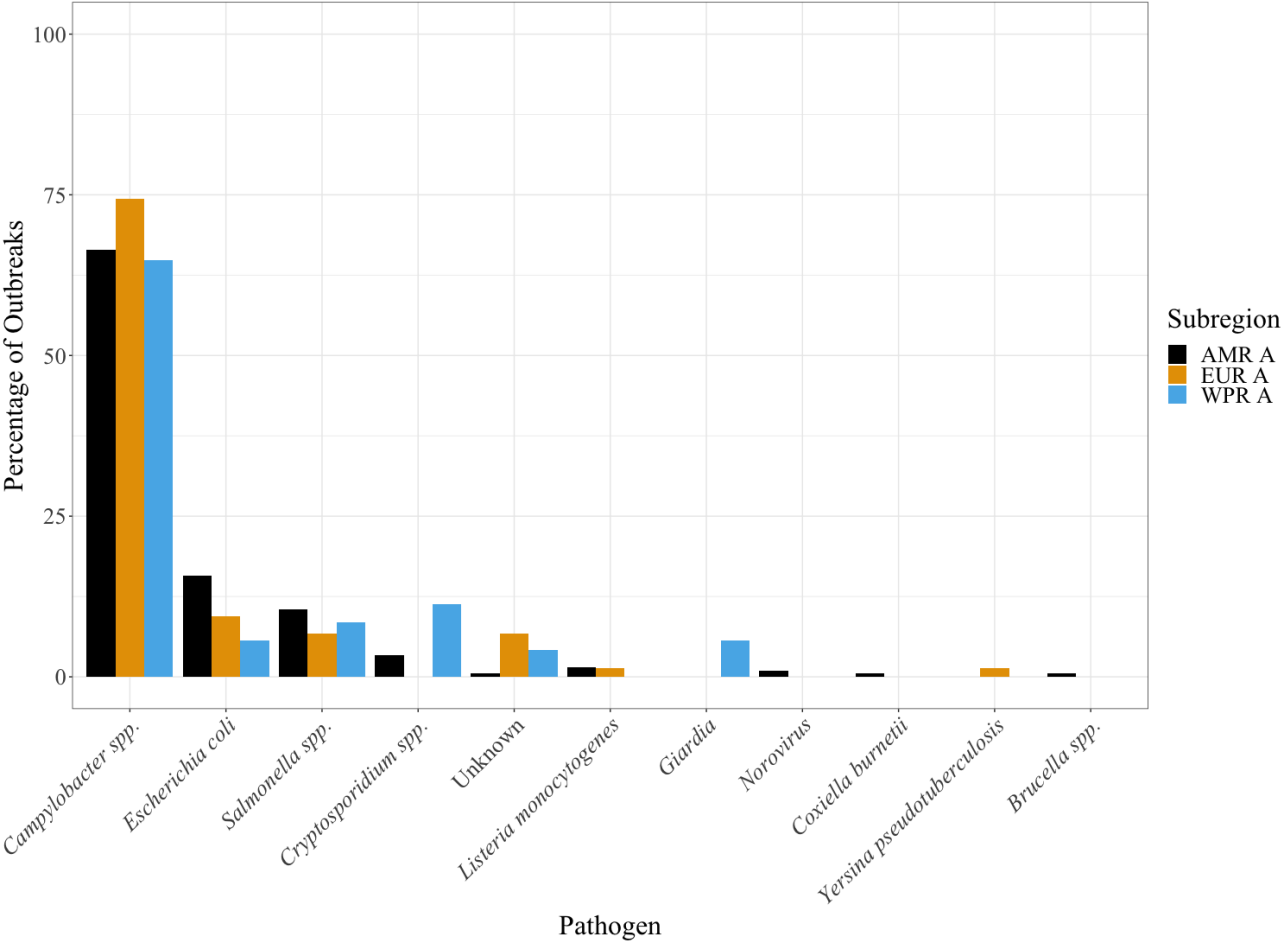
Histogram demonstrating the pathogens cultured in the different WHO subregions between the years 2000 and 2018. *Campylobacter* spp. were the most common. Further data by country is available in Table S2.

The aetiologies of these outbreaks did not differ geographically between WHO subregions, as shown in Fig. 3. *Campylobacter* spp. were the most common pathogens cultured (67.8%), followed by *E. coli* (12.4%) and *Salmonella* spp. (9.3%). Of the *Campylobacter* spp. cultures, *Campylobacter jejuni* accounted for 59.3% of the outbreaks; *Campylobacter coli* accounted for 1.7%; and the remainder were not identified at the species level.

**Figure 4:**
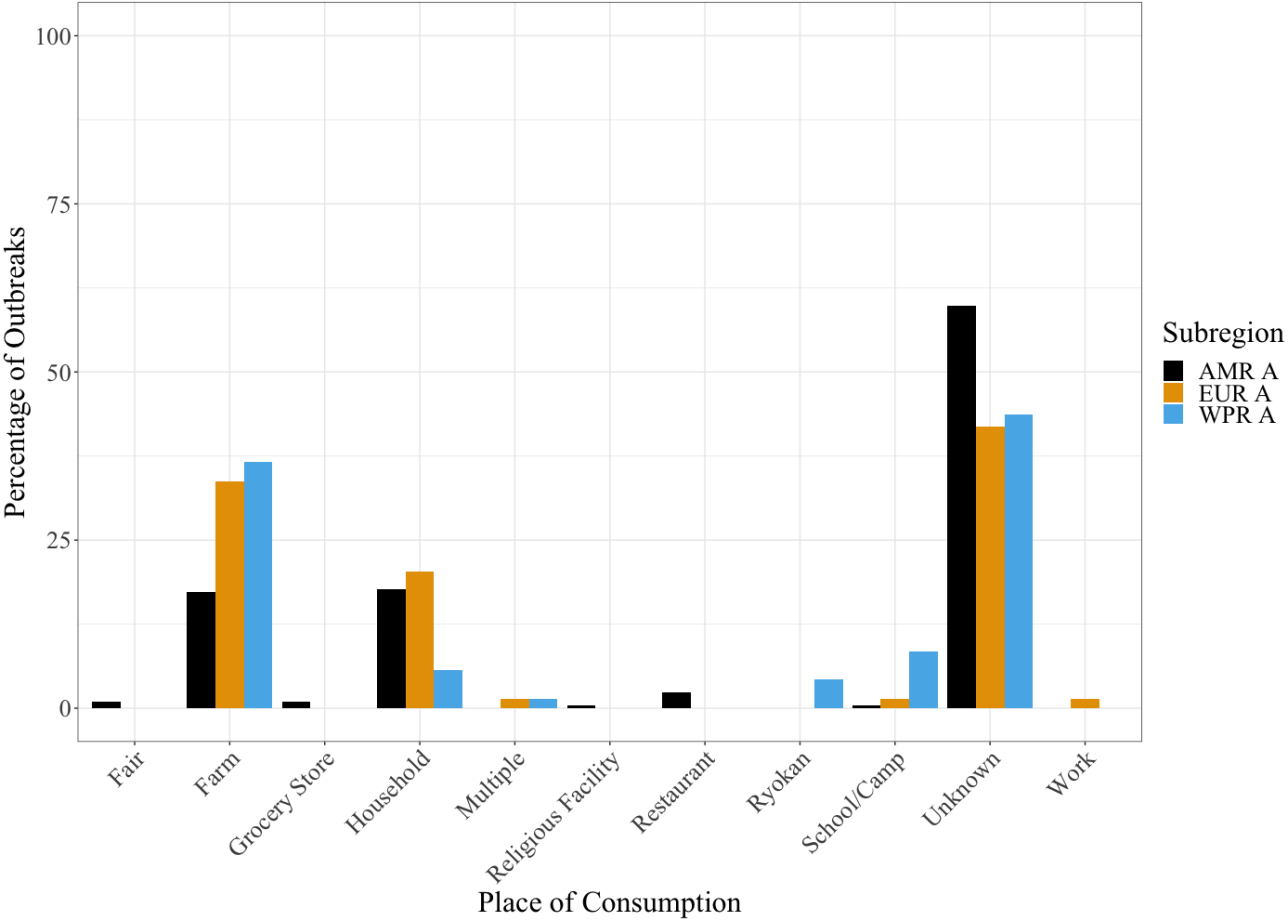
Histogram demonstrating the most common places of consumption of unpasteurised milk where data was available for the years 2000 to 2018. Farm and household consumption were the most common known sources globally. Further data by country is available in Table S3.

As seen in Fig. 4, most countries did not have any record of where the milk was consumed. However, of the outbreaks in which the source could be traced, farm consumption, followed by household consumption, were the most common locations. This finding was consistent geographically across WHO subregions.

## Discussion

The RER and outbreak data demonstrate that unpasteurised milk consumption remains a constant threat for foodborne disease across developed, high-income countries, despite the ready availability of safer, pasteurised milk. Illegal acquisition of milk remains a potential problem even in countries with strict regulations banning the sale of unpasteurised milk. In addition to the importance of state regulation, research and consumer education, other methods to decrease the risk need to be explored.

The outbreak data indicate that across all high-income countries, the aspects of unpasteurised milk outbreaks are similar in nature. Consumption often happened at the farm or in an individual’s household, regardless of which WHO subregion the outbreak occurred in. This is likely to be because in many countries where unpasteurised milk is legal, it can only be purchased from the farm gate or farm vending machine, rather than from a large-scale distributor. Nevertheless, this is a positive requirement, as it reduces the number of steps of the risk pathway, as seen in Fig. 1. The requirement for proactively going to the farm indicates that individuals usually choose to consume unpasteurised milk, rather than consuming it unknowingly from widespread distribution at social events or in community settings.

The responsible pathogens for the outbreaks analysed in this study were also similar in occurrence in all geographical regions. The pathogens observed in the data were very similar to those reported in the literature, apart from *Toxoplasma gondii*, *Mycobacterium bovis* and *Staphylococcus aureus,* which were not observed in the data obtained in this study. In addition to this, there were four outbreaks of *Giardia* and two outbreaks of *Norovirus* associated with unpasteurised milk consumption in the data, which, as far as the authors can determine, have not previously been reported in the literature for unpasteurised bovine liquid milk consumption.

At the time of this research, there were 80 countries classified as high-income by the World Bank, where the gross national income per capita exceeds $12,696 (World Bank, 2021). This study included 15 of those 80 countries, representing approximately 70% of the total population of all high-income countries. It is prudent to consider that regulations and population size are not the only variables affecting unpasteurised milk disease outbreaks–culture and volume of milk consumption also play a role. Dairy consumption varies across the world, and therefore if there was a country with a large population which allows unpasteurised milk, yet consumption of dairy is uncommon, there would be fewer outbreaks. Therefore, further research is required to be able to statistically compare regulations, population size and number of outbreaks reliably.

### Solutions to improving the safety of unpasteurised milk consumption

The NSW Government in Australia has taken a particularly strong position on the sale of illegal unpasteurised milk, where the Food Act states there may be penalties of up to $275,000 for the sale of unpasteurised milk (NSW Food Authority, 2014). As a result, there are very few unpasteurised milk-related outbreaks, and none since 2003. Conversely, there is the possibility that illnesses are not disclosed due to the fear of high penalties being enforced. While penalties are not a perfect solution, it may be an option for countries which have made the sale of unpasteurised milk illegal yet find that consumers are finding alternatives, such as the purchasing of ‘pet milk’, or buying into cow share schemes.

Nevertheless, even though dedicated consumers of unpasteurised milk may find alternative ways to acquire it, the FSA’s 2018 consumer survey in the UK found that 41% of consumers actually wanted government protection and regulation of unpasteurised milk (increased from 27% in 2012) (Food Standards Agency (FSA), 2018b).

While regulation is in place in most countries, particularly at the milk processing stage, it does not always mitigate the possibility of foodborne disease. For example, in the UK, Schedule 6 of the Food Safety and Hygiene (England) Regulations 2013 states that there must be compliance with the standards for total bacteria count (<20,000 cfu per ml) and coliforms (<100 cfu per ml), as well as the cows supplying the milk needing to be tuberculosis- and brucellosis-free (Food Standards Agency (FSA), 2018a). Despite these requirements, Willis et al. (2018) found that almost half of all unpasteurised milk samples tested in their study still contained pathogens, or indicators of poor hygiene were present. Nevertheless, it is possible for raw milk producers to meet very high standards of hygiene, which has been demonstrated by the producers of milk known as *Vorzugsmilch* (VZM) in Germany (Berge & Baars, 2020). The standards of this milk are tightly regulated by the German government and exceeding the upper limits of laboratory microbial tests will result in an immediate ban of sales and recall of all recently-sold milk.

Another option to improve safety is to increase pathogen testing. High-risk foods commonly are required to undergo ‘test and hold’ practices pending a negative result. Some member farms of the USA’s Raw Milk Institute, for example, have implemented on-farm testing for coliforms and total bacteria counts, which allows for quick ‘test and hold’ practices to be carried out (Berge & Baars, 2020). However, milk is highly perishable, and spoilage and pathogenic organisms may proliferate during the waiting time for the test results when sent to external laboratories (Food Standards Australia New Zealand (FSANZ), 2009). In addition to this, the most common pathogen—*Campylobacter* spp.—requires microaerophilic conditions to culture, which often makes it hard to detect. Nevertheless, real-time PCR testing has been shown to successfully detect *Campylobacter jejuni* and *Campylobacter coli* in 60 min (Wulsten, Galeev & Stingl, 2020), but the availability and financial implications of testing may not make it viable for many farmers. Therefore, unless this process can be accelerated and further developed, it is somewhat limited in its practicality for ensuring the safety of unpasteurised milk in the field.

There have also been some other processes which have been suggested as an alternative to pasteurisation, which improve the safety and shelf-life of the milk without major detriment to its structural or nutritional profile. This may make such milk more attractive to consumers who prefer unpasteurised milk, particularly consumers who choose to consume unpasteurised milk due to a taste preference. The most-used method for pasteurisation for countries in this study required high-temperature-short-time (HTST) conditions, which involves heating to 71−72 °C for 15 seconds, but varying the temperature and time can considerably alter the taste characteristics of the milk (Toko et al., 1995). Pascalisation, also known as high-pressure processing (HPP), is a process which uses 100–600 MPa, 1–6 kbar high pressure and does not heat the milk. It has been demonstrated that the effects of pascalisation include inactivation of microbes, however bioactive proteins remain intact (Chughtai et al., 2021). While this does decrease the number of bacteria present, it is not fully effective against Shiga-toxin producing *E. coli* (Office for Risk Assessment & Research (BuRO), 2017). In addition to this, the inactivation of the enzyme alkaline phosphatase in milk is what most countries require by law to prove that the milk has been sufficiently pasteurised, but pascalisation does not inactivate the enzyme, meaning it is impossible to prove that the milk has successfully gone through the process (Office for Risk Assessment & Research (BuRO), 2017).

Other suggested treatments for milk are pulsed electric field treatment, UV light, ultrasound, cold plasma treatment, micro fluidisation, infrared spectroscopy and membrane microfiltration (Office for Risk Assessment & Research (BuRO), 2017; Chughtai et al., 2021). In addition, clostridial spores can be targeted for removal via bactofugation and microfiltration, or inhibition through the addition of nitrate and lysozyme (Komori et al., 2019). For countries which allow the sale of unpasteurised milk, these treatments offer an opportunity to improve its safety. However, consumption of unpasteurised milk is ultimately at the consumer’s own risk. Better communication about the hazards associated with consuming unpasteurised milk is needed, particularly to high-risk consumers, such as the young, old, and clinically vulnerable (European Food Safety Authority (EFSA), 2015). This is best achieved through message clarity, repetition and delivery of the information from a credible source (Angulo, Lejeune & Rajala-Schultz, 2009).

Most consumer communication is presented in the form of labelling. However, this is not a legal requirement everywhere. In Spain, the label must state “Raw milk not heat-treated: boil before consumption” and “Store in refrigeration between 1 and 4 °C”. It has also been recommended that the shelf life is set at three days, and a warning is placed that there could be health risks (Spanish Agency for Food Safety and Nutrition (AESAN), 2020). Similarly, in the UK, the FSA requires the following label: “This milk has not been heat-treated and may therefore contain organisms harmful to health”. This has been updated to include: “The FSA strongly advises that it should not be consumed by children, pregnant women, older people and those who are unwell or have chronic illness”, but the additional wording is only required in Wales (Food Standards Agency (FSA), 2020).

However, in many countries, unpasteurised milk is sold in ways which allow consumers to legally circumvent restrictions, for example as ‘pet milk’. In these cases, appropriate safety labelling will not be in place. In 2008, in North Carolina, there was a proposed rule to have dye added to ‘pet milk’. However, this resulted in a lobbying campaign which overturned the rule (Whitehead & Lake, 2018). In Victoria, Australia, to prevent illegal consumption, unpasteurised milk must be tainted with a bitter gagging agent (Australian Institute of Food Safety, 2015).

There does not appear to be a perfect solution to improving the safety of unpasteurised milk consumption, however, as discussed, there have been some promising developments in the way unpasteurised milk can be gently or minimally processed to reduce microbial risk. Better risk communication strategies need to be implemented in countries which legalise the sale of unpasteurised milk, which include information about hazards, the importance of boiling before consumption, as well as the minimal effect pasteurisation has on milk’s nutritional quality. This will enable consumers to make an unbiased, informed decision whether drinking unpasteurised milk is right for them.

### Limitations

The results of this research should be regarded as indicative of the hazards of unpasteurised milk, as the data was incomplete for all high-income countries. Fifteen countries out of a possible of 80 provided data for the study, and a further nine countries were not specifically excluded, but did not record any outbreaks. In addition to this, for some of the countries which were cited, data was incomplete. For example, in 2009, the NORS outbreak data for the USA had some data quality issues, and the number of reports in that year decreased by approximately 50% compared to the previous 5-year average (Whitehead & Lake, 2018).

Another limitation of this research is that there is not standardisation in reporting systems, and they differ from one country to the next. Some countries have made almost all foodborne disease pathogens notifiable, therefore have a large dataset of outbreak information from both passive and active surveillance. Other countries do not report every outbreak, or do not collect specific information about the food vehicle involved, resulting in difficulties interpreting the true incidence of unpasteurised milk-associated disease outbreaks. In addition to that, very few countries record or report the volume of dairy consumption, particularly unpasteurised dairy, which presents the question, is dairy-borne disease risk related to: consumption of unpasteurised milk and milk products being simply an inherently risky practice; differences in milk hygiene standards or legislation between countries; or the volume of raw milk consumed? Nevertheless, the World Health Organisation has provided a resource for countries to standardise reporting procedures for foodborne outbreaks, which highlights unpasteurised milk as a food vehicle for most of the pathogens cultured in this study (World Health Organization (WHO), 2018).

Finally, to gain a more complete understanding of the drivers, benefits and hazards of unpasteurised milk and milk product consumption in high-income countries on a global scale, a full systematic review would be beneficial, as a rapid evidence review will not as thoroughly explore all the literature available, and the findings are indicative, but useful nonetheless.

### Further studies

This research provides a preliminary source of information regarding the drivers and hazards across high-income countries. There is an increasing demand for unpasteurised milk in high-income countries, and further studies into the alternatives to pasteurisation would address a research gap in the literature. Since the most common reason cited for drinking unpasteurised milk was for health benefits, particularly the belief that it has a higher nutritional content, this research should aim to better understand how the milk is altered through pasteurisation, to provide a focus point for the development of techniques which further improve food safety.

Ultimately, it is not fully understood which unpasteurised milk regulations have the highest impact on the number of foodborne disease outbreaks, nor whether regulations have a significant protective impact. Further studies need to more fully explore what other variables impact the number of outbreaks associated with unpasteurised milk, as well as quantify the amount of unpasteurised milk consumption per country. This information could reveal the best approach for countries to adopt in order to better manage the human health risks associated with the consumption of unpasteurised milk and milk products.

## Conclusions

People consume unpasteurised milk due to several observable health benefits, including reduced risk of asthma, atopy, and respiratory conditions, as well as other perceived health benefits which may not have been as rigorously studied, such as improved digestion, reduced risk of rectal cancer and improved nutritional quality. Consumer advocates may simply prefer the taste of unpasteurised milk and associated products, or are keen to support local producers at source, with a view to environmental and social sustainability. However, this may come with a cost, as foodborne disease outbreaks associated with consumption of unpasteurised milk and associated products are on the rise in high-income countries, and are most commonly associated with *Campylobacter* spp., *E. coli* and *Salmonella* spp.

It cannot definitively be concluded whether the benefits of unpasteurised milk outweigh the hazards, as they are likely to be different for every individual. For example, a child drinking unpasteurised milk may have a smaller chance of developing asthma or atopy, yet a greater chance of acquiring a life-threatening infection from unpasteurised milk, compared to an adult consumer. The elderly and the immunocompromised are also more likely to suffer ill effects from unpasteurised milk consumption. Apart from the potential benefits, there are some very serious public health hazards associated with the consumption of unpasteurised milk which need to be more effectively communicated to consumers in order that their decisions to drink it regardless of legislation or advice are fully informed. Views will differ on the role of state regulation *versus* individual responsibility and the freedom to make informed choices, and stakeholders such as primary producers, veterinarians, nutritionists and microbiologists may differ markedly in their opinions on the balance between benefits and harms. The ultimate responsibility arguably lies with the consumer to protect their own health, and regulations are there for further protection as well as strongly influencing consumer behaviour. Regardless, many people choose to continue to consume unpasteurised milk despite the known health risks from primarily biological hazards, therefore further research into treatment alternatives to pasteurization and improvement of hygiene standards at farm level are required so that a safer product can consistently be made available.

##  Supplemental Information

10.7717/peerj.13426/supp-1 Supplemental Information 1 The number of dairy foodborne outbreaks by country for the period of 2000–2018 The raw data for Canada is not publicly available but can be requested from the Public Health Agency of Canada directly. Some of this data was obtained directly from government agencies, and some is publicly available from academic articles or public online repositories. The source is detailed in the last column.

10.7717/peerj.13426/supp-2 Supplemental Information 2 The percentage of dairy foodborne outbreaks associated with common pathogens by country for the period of 2000–2018 The raw data for Canada is not publicly available but can be requested from the Public Health Agency of Canada directly. Some of this data was obtained directly from government agencies, and some is publicly available from academic articles or public online repositories. The source is detailed in the last column.

10.7717/peerj.13426/supp-3 Supplemental Information 3 The percentage of dairy foodborne outbreaks associated with different places of consumption by country for the period of 2000–2018 The raw data for Canada is not publicly available but can be requested from the Public Health Agency of Canada directly. Some of this data was obtained directly from government agencies, and some is publicly available from academic articles or public online repositories. The source is detailed in the last column.

10.7717/peerj.13426/supp-4 Supplemental Information 4 Raw dataset containing outbreak information

## Additional Information and Declarations

### Data Availability

The following information was supplied regarding data availability: The raw data, outbreak information of food borne diseases relating to raw milk consumption, is available in the Supplemental File.
