# Supplementary material for: Drivers and hazards of consumption of unpasteurised bovine milk and milk products in high-income countries
Source: PeerJ. 2022 May 16;10:e13426. doi: 10.7717/peerj.13426 (PMC9135038; doi:10.7717/peerj.13426)
Supplement: Supplemental Information 2 — The raw data for Canada is not publicly available but can be requested from the Public Health Agency of Canada directly. Some of this data was obtained directly from government agencies, and some is publicly available from academic articles or public online repositories. The source is detailed in the last column. [file peerj-10-13426-s002.docx]

| Countries | *Brucella* | *Campylobacter* spp*.* | *Coxiella burnetii* | *Cryptosporidium* spp*.* | *Escherichia coli* | *Giardia* | *Listeria monocytogenes* | *Norovirus* | *Salmonella* spp*.* | Unknown | *Yersina pseudotuberculosis* |
| --- | --- | --- | --- | --- | --- | --- | --- | --- | --- | --- | --- |
| USA | 0.28% | 39.27% | 0.28% | 1.98% | 8.47% | 0.00% | 0.85% | 0.00% | 6.21% | 0.28% | 0.00% |
| New Zealand | 0.00% | 10.73% | 0.00% | 1.98% | 1.13% | 1.13% | 0.00% | 0.00% | 1.41% | 0.56% | 0.00% |
| Germany | 0.00% | 10.45% | 0.00% | 0.00% | 0.28% | 0.00% | 0.00% | 0.00% | 0.00% | 0.00% | 0.00% |
| UK | 0.00% | 1.13% | 0.00% | 0.00% | 0.85% | 0.00% | 0.28% | 0.00% | 0.28% | 1.41% | 0.00% |
| Finland | 0.00% | 1.13% | 0.00% | 0.00% | 0.28% | 0.00% | 0.00% | 0.00% | 0.00% | 0.00% | 0.28% |
| Australia | 0.00% | 1.13% | 0.00% | 0.28% | 0.00% | 0.00% | 0.00% | 0.00% | 0.00% | 0.28% | 0.00% |
| Denmark | 0.00% | 1.41% | 0.00% | 0.00% | 0.00% | 0.00% | 0.00% | 0.00% | 0.00% | 0.00% | 0.00% |
| Japan | 0.00% | 1.13% | 0.00% | 0.00% | 0.00% | 0.00% | 0.00% | 0.00% | 0.28% | 0.00% | 0.00% |
| Netherlands | 0.00% | 0.85% | 0.00% | 0.00% | 0.28% | 0.00% | 0.00% | 0.00% | 0.00% | 0.00% | 0.00% |
| Austria | 0.00% | 0.28% | 0.00% | 0.00% | 0.00% | 0.00% | 0.00% | 0.00% | 0.28% | 0.00% | 0.00% |
| Ireland | 0.00% | 0.00% | 0.00% | 0.00% | 0.28% | 0.00% | 0.00% | 0.00% | 0.28% | 0.00% | 0.00% |
| Sweden | 0.00% | 0.28% | 0.00% | 0.00% | 0.00% | 0.00% | 0.00% | 0.00% | 0.00% | 0.00% | 0.00% |
| France | 0.00% | 0.00% | 0.00% | 0.00% | 0.00% | 0.00% | 0.00% | 0.00% | 0.28% | 0.00% | 0.00% |
| Croatia | 0.00% | 0.00% | 0.00% | 0.00% | 0.00% | 0.00% | 0.00% | 0.00% | 0.28% | 0.00% | 0.00% |
